# Advances in the Diagnosis of Rheumatoid Arthritis-Associated Interstitial Lung Disease: Integrating Conventional Tools and Emerging Biomarkers

**DOI:** 10.3390/ijms27031165

**Published:** 2026-01-23

**Authors:** Jing’an Bai, Fenghua Yu, Xiaojuan He

**Affiliations:** Institute of Basic Research in Clinical Medicine, China Academy of Chinese Medical Sciences, Beijing 100700, China; baijingancatcm@163.com (J.B.); fenghua_yy@163.com (F.Y.)

**Keywords:** rheumatoid arthritis-associated interstitial lung disease, diagnosis, high-resolution computed tomography, artificial intelligence, biomarkers

## Abstract

Rheumatoid arthritis-associated interstitial lung disease (RA-ILD) is one of the most common extra-articular manifestations of rheumatoid arthritis (RA) and a leading cause of mortality in RA patients. The diverse and nonspecific clinical presentations of RA-ILD make early diagnosis particularly challenging. In recent years, with a deeper understanding of the pathogenesis of RA-ILD and rapid advancements in medical imaging, artificial intelligence (AI) technologies, and biomarker research, notable progress has been achieved in the diagnostic approaches for RA-ILD. This review summarizes the latest research developments in the diagnosis of RA-ILD, with a focus on the clinical practice guidelines released in 2025. It discusses the application of high-resolution computed tomography (HRCT), the potential of AI in assisting HRCT-based diagnosis, and the discovery and validation of biomarkers. Furthermore, the review addresses current diagnostic challenges and explores future directions, providing clinicians and researchers with a cutting-edge perspective on RA-ILD diagnosis.

## 1. Introduction

Rheumatoid arthritis (RA), a chronic systemic inflammatory disease characterized primarily by symmetric polyarthritis, has implications that extend far beyond the synovium and periarticular tissues [1,2]. As a systemic disorder capable of affecting multiple organ systems, the lungs represent one of the most common sites of extra-articular involvement, with pulmonary complications posing serious threats to patient outcomes. Among these pulmonary manifestations, interstitial lung disease (ILD) is particularly prominent. It not only constitutes a significant contributor to increased morbidity and mortality in RA patients but also presents substantial challenges in clinical diagnosis and management [3,4,5]. The pathology of RA-ILD involves complex inflammation and fibrosis of the pulmonary interstitium, which can lead to irreversible lung function loss and respiratory failure [6,7]. Consequently, the early identification and accurate diagnosis of RA-ILD are crucial for improving the overall prognosis and quality of life for RA patients. However, the clinical presentation of RA-ILD is highly variable and non-specific, ranging from subclinical disease detectable only by imaging to severe dyspnea and cough [8,9,10]. This wide spectrum of manifestations, coupled with the fact that lung involvement can precede or follow joint symptoms by years, makes establishing unified diagnostic criteria challenging [11,12]. Although high-resolution computed tomography (HRCT) is considered the “gold standard” for detecting radiologic lung changes in RA-ILD, and pulmonary function tests (PFTs) are vital tools for monitoring disease progression, clinical practice still faces considerable confusion regarding when to initiate screening, how to interpret results, and how to integrate multifaceted clinical information [8,13]. Current research on the diagnosis of RA-ILD lies at the convergence of rheumatology, pulmonology, molecular biology, bioinformatics, and imaging sciences.

Recent years have seen progress in this field, fueled by a deeper understanding of pathogenesis and new technologies. For instance, the application of artificial intelligence (AI) in medical image analysis has opened new avenues for the precise diagnosis and quantitative assessment of RA-ILD. Furthermore, research into biomarkers is gaining increasing attention, holding promise for providing new clues for the early diagnosis, disease assessment, and prognosis prediction of RA-ILD [14,15]. This review summarizes the latest advances in the diagnosis of RA-ILD. In contrast to previous reviews, this one not only synthesizes the current status of conventional diagnostic tools (HRCT, PFTs) and their roles in the latest guidelines but also critically examines the paradigm shift in practice brought about by the 2025 clinical practice guidelines from the European Respiratory Society (ERS) and the European Alliance of Associations for Rheumatology (EULAR). It further analyzes the real-world challenges and validation gaps in AI-assisted imaging quantification and reviews the current status and development bottlenecks of biomarkers. It aims to provide readers with a timely and critical reference.

## 2. Epidemiology of RA-ILD

Reported rates of RA-ILD vary considerably across studies due to the heterogeneity in populations, diagnostic criteria, and study designs [16]. RA-ILD imposes a substantial burden of morbidity and mortality, representing a leading cause of death, particularly among the elderly [16,17]. For example, a large US Medicare cohort of elderly RA patients (*N* = 509,787; mean age 72.6 years) found a baseline RA-ILD prevalence of 2.0%, with an additional 2.6% developing it over a median 3-year follow-up, yielding a cumulative incidence of nearly 5% [18]. Another systematic review and meta-analysis focusing on CTD-ILD reported a pooled prevalence of ILD in RA patients of 11% [19]. However, other studies have reported higher prevalence rates, reflecting differences in study populations and diagnostic methods [20,21]. For instance, screening asymptomatic RA patients using HRCT may identify a higher proportion of subclinical ILD, particularly among those who are ACPA-positive and exhibit abnormalities in physiological tests such as PFTs or cardiopulmonary exercise testing [21]. RA-ILD is associated with multiple factors, and identifying these high-risk factors is crucial for early diagnosis and intervention. Advanced age is a key risk factor for RA-ILD onset, progression, and mortality. A meta-analysis showed increased mortality risk with age (HR = 1.04) [17], and the progression risk rises notably after 65 [22]. Male sex is associated with higher mortality (HR = 1.44) [17] and a greater propensity for the poor-prognosis UIP pattern [23]. Smoking is another well-established risk factor, not only increasing the risk of developing RA but also significantly elevating the risk of RA-ILD. A strong association has been confirmed between heavy smoking (e.g., ≥30 pack-years) and RA-ILD (OR 6.06, 95% CI 2.72–13.5) [24,25]. Genetic factors also play an important role in the pathogenesis of RA-ILD, with particular attention on the MUC5B rs35705950 polymorphism [26]. The T allele of this gene is associated with an increased risk of idiopathic pulmonary fibrosis (IPF) and other fibrotic ILDs, and further research has demonstrated that carriers of the rs35705950 T allele have a significantly elevated lifetime risk of RA-ILD [27]. Serological markers, such as positivity for rheumatoid factor (RF) and anti-citrullinated protein antibodies (ACPA), as well as high ACPA titers, are also associated with an increased risk of RA-ILD [28]. ACPA positivity is not only associated with the occurrence of RA-ILD but may also correlate with more severe lung involvement and a poorer prognosis [28]. High RA disease activity itself, indicated by measures like a high DAS28 score, is also recognized as a risk factor for RA-ILD [29]. Furthermore, some studies have found that obesity (OR 2.42, 95% CI 1.11–5.24 compared to normal weight), elevated C-reactive protein (CRP) levels (≥10 mg/L vs. <3 mg/L, OR 2.61, 95% CI 1.21–5.64), and poor functional status (e.g., Multidimensional Health Assessment Questionnaire score ≥ 1 vs. <0.2, OR 3.10, 95% CI 1.32–7.26) may be associated with an increased risk of RA-ILD [24].

Integrating these risk factors, researchers have attempted to develop risk score models to predict the occurrence of RA-ILD. For example, one study combining lifestyle and clinical risk factors achieved an AUC of 0.79 (95% CI 0.73–0.85) for predicting RA-ILD [24]. While the predictive performance of the model has room for improvement, they offer the potential for selective screening of high-risk RA patients. Early screening criteria for RA-ILD proposed via Delphi methods recommend selective screening based on clinical risk factors [30].

## 3. The Evolution of Diagnostic Criteria and Guidelines: Core Recommendations of the 2025 ERS/EULAR CTD-ILD Clinical Practice Guidelines

For a long time, the diagnosis of RA-ILD has largely relied on the empirical judgment of clinicians [31]. In recent years, with the deepening understanding of the pathogenesis of RA-ILD and the continuous accumulation of high-quality clinical evidence, the international medical community has gradually promoted the formulation of unified clinical practice guidelines to standardize the diagnosis and treatment pathways of RA-ILD [32,33]. Among them, the 2025 ERS/EULAR CTD-ILD clinical practice guidelines, as the first cross-society and cross-specialty comprehensive clinical practice guidelines for CTD-ILD, provide relatively comprehensive recommendations for the screening, diagnosis, and monitoring of CTD-ILD, including RA-ILD [34]. The core recommendations can be summarized as follows: Regarding screening strategies, the guidelines emphasize the central role of HRCT and explicitly state that using only PFTs or Lung Ultrasound (LUS) to replace HRCT for ILD screening is not recommended [34]. This is primarily because HRCT demonstrates higher sensitivity and specificity in detecting early and mild ILD lesions. Additionally, the guidelines also stress the importance of individualized screening, meaning that high-risk populations requiring screening should be identified based on specific risk factors associated with different CTDs, avoiding indiscriminate screening for all patients. In the domain of diagnosis, the guidelines highlight the value of multidimensional risk assessment and restrict the applicable scenarios for bronchoalveolar lavage (BAL) and lung biopsy [34]. This approach indicates that for typical RA-ILD cases, a clinical diagnosis can often be made by integrating clinical manifestations, serological markers (such as ACPA and RF), and characteristic HRCT findings, without routinely performing invasive procedures like BAL or lung biopsy [35]. With respect to monitoring, the guidelines specify the intervals for PFTs and HRCT re-examinations for different CTD-ILD patients, emphasizing the need to balance disease activity and progression risk [34]. The guidelines advocate for a risk assessment-based strategy to screen for ILD in patients with RA, signifying a substantial transition from a uniform screening approach toward precision medicine. In contrast to earlier clinical practice guidelines or expert proposal, the updated guidelines incorporate risk factors more systematically—accounting for elements such as MUC5B genotype—and thereby embody a paradigm shift from blanket screening to targeted, risk-stratified screening [30,36]. However, this evolution also brings several meaningful gaps and unresolved questions into sharp relief. A considerable proportion of recommendations still rely on observational studies or expert consensus, reflecting evidence limitations. Consequently, critical implementation gaps remain. First and foremost, guidance on operationalizing risk stratification is unclear: which prediction models to use and how to categorize patients into risk tiers? Furthermore, practical questions about the screening pathway persist, including the choice of sequential screening tools and defining evidence-based screening intervals for different risk groups. In addition, given the limitations of the current evidence, the guidelines do not delineate a formal role for artificial intelligence in screening and diagnostic processes, leaving its integration into clinical workflows as an open question.

## 4. Clinical Application of Conventional Diagnostic Tools: HRCT and PFTs

### 4.1. Clinical Application of HRCT in RA-ILD

#### 4.1.1. Advantages and Limitations of HRCT

In RA-ILD, the most common HRCT patterns are UIP and NSIP, followed by less common patterns such as organizing pneumonia (OP) and lymphoid interstitial pneumonia (LIP) [37,38]. Different patterns present with characteristic imaging findings and are closely associated with prognosis and treatment options [37,39,40,41,42].

HRCT enables both RA-ILD detection and pattern classification, which is vital for prognosis and treatment planning [43,44]. For example, a UIP pattern may indicate potential benefit from antifibrotic therapy [42]. It also allows for semi-quantitative assessment of lesion extent and severity [45]. However, interpretation can be subjective, with variable inter-observer agreement, especially for atypical or mild cases [46]. While central to diagnosis, the 2025 ERS/EULAR guidelines recommend individualized decision-making for screening asymptomatic patients, considering radiation exposure risks [34]. Table 1 summarizes the key imaging characteristics of these patterns.

#### 4.1.2. Current Status and Prospects of AI-Assisted HRCT Diagnosis

Integrating artificial intelligence (AI) into RA-ILD diagnosis aims to augment subjective radiological interpretation with objective, data-driven quantification. AI technologies, particularly deep learning, have indeed made progress in medical image analysis and begun to be applied in ILD diagnosis [49,50]. AI applications span the diagnostic continuum. At the acquisition level, deep learning reconstruction (DLR) algorithms are demonstrating the capacity to generate images of enhanced quality from raw projection data, achieving notable reductions in noise and improvements in spatial resolution. Critically, this is accomplished without an increase in radiation dose, and early evidence suggests diagnostic fidelity can be maintained even within ultra-low-dose protocols—a feature of particular value for the serial imaging required in chronic disease management [51,52]. Concurrently, advancements in CT hardware also contribute by providing higher-resolution input data for these algorithms [53,54]. The foundational output of this enhanced imaging is leveraged by advanced convolutional neural networks for automated quantitative analysis. Models based on architectures like nnU-Net exhibit high precision in the semantic segmentation of cardinal ILD patterns, including ground-glass opacities, reticulation, and honeycombing. This process effectively transmutes qualitative visual assessments into precise, continuous, and reproducible imaging biomarkers [55]. Beyond volumetric quantification, the field is exploring higher-order analytical capabilities. Radiomics, which entails the high-throughput extraction of sub-visual texture and shape features, is being investigated for its potential to discern biologically relevant phenotypes, such as distinguishing predominantly fibrotic from inflammatory patterns, which could inform more nuanced therapeutic decisions [55,56]. AI models extracting these features hold promise not only for assessing current severity but also for predicting disease progression and treatment response [57,58]. Concurrently, decision-support tools like content-based image retrieval (CBIR) systems are being developed to function as intelligent reference archives. By allowing clinicians to query a database with an image region of interest, these systems can retrieve cases with analogous imaging manifestations and their corresponding diagnoses, thereby serving as an adjunct for pattern recognition and differential diagnosis [59].

Despite these substantive technical advancements, the collective evidence base has not yet matured to a level that warrants the routine clinical adoption of AI tools for RA-ILD diagnosis. The current status is perhaps most clearly demarcated by the position of authoritative guidelines. The 2024 European Respiratory Society statement advocates for quantitative CT metrics but refrains from endorsing specific AI-based software for diagnostic application [50]. This critical distinction underscores a prevailing consensus: while the principles of quantification are increasingly embraced, the specific AI implementations remain in a phase of validation and evidence accrual. Consequently, these technologies are presently regarded as powerful investigational tools with considerable prospective utility, rather than as standardized components of established diagnostic protocols.

Translating AI research into clinical utility faces several challenges. A primary concern lies in the developmental provenance of many published models, which are predominantly derived from retrospective, single-center datasets of limited scale. This paradigm carries an inherent risk of overfitting and “shortcut learning,” wherein models may inadvertently codify institution-specific scanning protocols, patient selection biases, or annotator idiosyncrasies, thereby compromising their generalizability to broader, more heterogeneous populations [49]. The performance metrics reported in such internal validation studies are therefore of uncertain external validity. This issue is compounded by a pronounced scarcity of robust, multi-center external validation studies. For AI in RA-ILD, comprehensive external validation remains exceptionally rare. Instructive parallels from other domains, such as mammography, demonstrate that model performance can degrade substantially in external settings, highlighting the tenuous nature of claims predicated solely on internal validation [60].

Finally, implementation faces systemic barriers. The pervasive “black-box” nature of complex deep learning models engenders a significant trust deficit among clinicians, as the opacity of the decision-making process complicates clinical reasoning and raises substantive ethical and medico-legal questions regarding accountability [61,62]. Concurrently, the development of robust and generalizable models is fundamentally constrained by the scarcity of large-scale, high-quality, and expertly annotated multi-institutional datasets. Data silos, privacy regulations, and the resource-intensive nature of annotation pose formidable obstacles to the collaborative data-sharing ecosystems that are essential for progress [63]. The integration of AI tools into existing hospital radiology information systems and clinical workflows without disrupting efficiency or adding cognitive load presents a further layer of socio-technical complexity. Moreover, the global regulatory frameworks for approving AI-based software as a medical device, alongside the evolving policies for reimbursement, remain in a state of flux, creating an environment of uncertainty for developers and healthcare institutions alike.

In conclusion, AI-assisted HRCT harbors transformative potential for RA-ILD by enabling a shift from impressionistic assessment to precise, quantitative phenotyping. However, realizing this potential requires moving from technical feasibility to proven clinical utility and overcoming significant implementation hurdles.

### 4.2. Clinical Application of PFTs in RA-ILD

PFTs serve as an important ancillary means for assessing the degree of respiratory impairment and monitoring disease progression in RA-ILD patients. Even in RA patients without respiratory symptoms, PFTs may detect underlying lung disease, and their use for screening asymptomatic patients is considerably more valuable than chest X-ray [64]. The 2025 ERS/EULAR clinical practice guidelines for CTD-ILD recommend regular PFT monitoring, with the specific frequency to be individualized [34]. The key roles and limitations of PFTs in the context of RA-ILD are systematically summarized in Table 2.

## 5. Research Advances in Emerging Biomarkers for the Diagnosis of RA-ILD

Conventional diagnostic tools such as HRCT and PFTs still present limitations in early diagnosis, monitoring disease activity, predicting treatment response, and distinguishing between different pathological subtypes. Ideal biomarkers should exhibit high sensitivity and specificity, be obtainable through minimally or non-invasive methods (such as blood tests), and aid in guiding clinical decision-making [74,75]. In recent years, with the rapid advancement of technologies in proteomics, genomics, and immunology, a series of emerging biomarkers for RA-ILD have been successively discovered and investigated. Table 3 provides a comparative analysis of emerging biomarkers, detailing their pathophysiological roles, advantages, limitations, and evidence levels. The following sections detail the biological rationale and key research findings for each category.

### 5.1. Biomarkers Associated with Alveolar Epithelial Cell Injury

Krebs von den Lungen-6 (KL-6) and Surfactant Protein D (SP-D) are two serum biomarkers that have been relatively well-studied and are considered to hold significant clinical potential [15,89]. Primarily derived from alveolar epithelial cells or involved in the process of pulmonary interstitial remodeling, elevated serum levels of these biomarkers are associated with alveolar epithelial injury, thus conferring value for the diagnosis and disease assessment of RA-ILD [79].

KL-6 is a glycoprotein expressed by proliferating, damaged, or regenerating type II alveolar epithelial cells [90]. Its serum levels rise when the alveolar architecture is disrupted. Multiple studies have confirmed that serum KL-6 levels are significantly higher in RA-ILD patients compared to RA patients without ILD and healthy control subjects [91,92]. A study reported high accuracy for KL-6 in diagnosing RA-ILD [76]. More importantly, serum KL-6 levels show a negative correlation with pulmonary function parameters (such as FVC and DLCO), suggesting that KL-6 levels can not only aid in diagnosis but also reflect the severity of ILD [77]. This indicates that dynamic monitoring of KL-6 may hold significant value for predicting disease progression.

SP-D, a key component of pulmonary surfactant, primarily functions in lung tissue. By binding to lipids, it regulates the metabolism and distribution of pulmonary surfactant, helping maintain alveolar physical stability and preventing alveolar collapse or overinflation [93,94]. Furthermore, as a crucial host defense protein in the lungs, SP-D specifically binds to carbohydrate motifs on pathogen surfaces, and while enhancing the phagocytic function of macrophages and neutrophils, it modulates inflammatory responses to prevent excessive immune damage [95,96]. When the integrity of the alveolar epithelium is compromised, SP-D leaks into the systemic circulation, leading to elevated serum SP-D levels [97]. Moreover, serum SP-D levels are significantly elevated in RA-ILD patients compared to RA patients without ILD and are correlated with disease severity, suggesting that SP-D might be an important predictive factor for RA-ILD progression [98,99].

### 5.2. Pulmonary Fibrosis-Related Biomarkers

Matrix metalloproteinase-7 (MMP-7) is a member of the matrix metalloproteinase family capable of degrading various extracellular matrix components such as elastin, fibronectin, and proteoglycans, playing a significant role in tissue remodeling and fibrosis [80,100,101]. In RA, inflammatory factors can promote MMP-7 expression, accelerating fibrosis and potentially contributing to RA-ILD development [81]. Studies have shown that MMP-7 expression is upregulated in various fibrotic lung diseases, including IPF, and is considered a biomarker of pulmonary fibrosis [82]. In RA-ILD, researchers have also found elevated serum MMP-7 levels in patients, which correlate negatively with pulmonary function parameters (e.g., FVC), suggesting an association with disease severity [79]. A systematic review and meta-analysis demonstrated that serum MMP-7 levels were significantly higher in RA-ILD patients compared to RA patients without ILD [15].

Periostin, a matricellular protein secreted by activated fibroblasts, is critically involved in pulmonary fibrosis pathogenesis. Primarily, periostin deposits within inflammatory and fibrotic foci, where it contributes to the sustenance of the inflammatory response [83]. Furthermore, it directly modulates fibroblast function and its expression is positively correlated with key fibrosis-related markers, including α-smooth muscle actin (α-SMA), type I collagen, TGF-β1, and fibronectin [84]. Furthermore, periostin promotes the differentiation of fibroblasts into myofibroblasts; studies demonstrate that genetic ablation of periostin suppresses α-SMA expression, while its pharmacological inhibition significantly impairs characteristic myofibroblast activities, such as collagen gel contraction and migratory capacity [84]. Collectively, these findings position periostin as an active effector molecule in the fibrotic cascade, propelling the progression of pulmonary fibrosis through its regulation of fibroblast function and facilitation of myofibroblast transformation.

Furthermore, within the context of RA-ILD, periostin shows promising potential as a biomarker for diagnosis. A prospective study measuring serum levels of monomeric and total periostin in RA-ILD patients, RA patients without ILD, and healthy controls found that periostin levels were significantly higher in the RA-ILD group compared to the other two groups [76]. Crucially, periostin levels correlate positively with the extent of fibrotic lesions on HRCT but not with inflammatory areas, suggesting it may specifically reflect the degree of pulmonary fibrosis [76]. In contrast, serum levels of biomarkers such as KL-6, SP-D, and lactate dehydrogenase (LDH) showed no significant correlation with the extent of these fibrotic areas.

### 5.3. Genetic and Cellular Senescence Biomarkers

In recent years, researchers have begun focusing on the roles of genetic background and cellular senescence in the pathogenesis and progression of RA-ILD, exploring related biomarkers. Among these, alterations in telomere length and the expression of associated proteins are recognized as important markers of cellular senescence and are linked to the risk of developing various interstitial lung diseases, including IPF, as well as disease severity [102,103]. Therefore, studying the association between telomere length and RA-ILD may offer new perspectives for understanding its pathogenesis and identifying high-risk populations.

Telomeres are specialized nucleotide repeat sequences located at the ends of chromosomes. They act like “caps” protecting chromosomes from degradation and fusion, thereby maintaining genomic stability [104]. With each cell division, telomeres progressively shorten due to the “end-replication problem” of DNA polymerase. When telomeres shorten to a critical length, they can trigger cell cycle arrest, cellular senescence or apoptosis [85]. The maintenance of telomere length primarily depends on the activity of the enzyme telomerase. Telomere-associated proteins, such as telomeric repeat-binding factor 1 (TERF1), are crucial regulators of telomere structure and function, and their expression levels correlate with telomere length. Given that telomere shortening is a well-established risk factor for IPF, researchers hypothesize that telomere dysfunction might also contribute to the pathogenesis of RA-ILD. Several studies have compared telomere length (TL) and TERF1 expression levels in peripheral blood mononuclear cells between RA-ILD patients, RA patients without ILD (RA-non-ILD), and healthy controls. The results showed that the overall TL in RA patients was significantly shorter than in healthy controls [86]. More importantly, TL in RA-ILD patients was also significantly shorter than in RA-non-ILD patients. Even after adjusting for sex, age, and disease duration, TL remained shorter in the RA-ILD group compared to the RA-non-ILD group. Further analysis revealed that when patients were grouped according to TL, the prevalence of ILD was significantly higher in the group with shorter TL compared to those with normal TL [86]. This suggests that telomere shortening may be associated with an increased risk of developing RA-ILD and could serve as a potential risk factor for ILD occurrence in RA patients. Furthermore, among RA-ILD patients, TL showed a negative correlation with disease duration, suggesting that telomeres might shorten further as the disease progresses, or that patients with poorer telomere function are more susceptible to developing or progressing to ILD.

Regarding TERF1 expression, studies found that TERF1 expression levels were significantly lower in RA patients (both with and without ILD) compared to healthy controls, and TERF1 expression levels positively correlated with TL. As a key regulator of telomere length and function, might reflect a general telomere dysfunction prevalent in RA patients [86].

While the mechanism by which telomere shortening leads to cellular senescence in the context of RA-ILD is not fully understood, it is thought to involve several interconnected pathways. A central proposed mechanism is that telomere shortening and senescence in alveolar epithelial cells impair their repair capacity and increase susceptibility to injury, thereby promoting fibrosis. Beyond structural cells, telomere shortening and senescence in immune cells may lead to immune dysregulation, characterized by increased secretion of pro-inflammatory cytokines—a phenomenon known as the senescence-associated secretory phenotype (SASP)—which can potentially exacerbate pulmonary inflammation and fibrosis. Compounding these effects, RA itself, as a chronic inflammatory disease, involves persistent immune system activation that might further accelerate immune cell senescence and telomere shortening. Furthermore, genetic factors, such as variations in telomerase-related genes, could predispose some RA patients to telomere dysfunction. Collectively, the interplay of these factors is believed to significantly increase the risk of developing ILD in RA patients [85,86,87,105].

Currently, research on the association between telomere length and RA-ILD is still relatively limited. Furthermore, issues such as telomere length measurement methods, tissue specificity (e.g., peripheral blood vs. lung tissue), and dynamic changes warrant further investigation. If telomere shortening is confirmed as an independent risk factor or prognostic marker for RA-ILD, it could help identify high-risk subgroups for ILD among RA patients, enabling closer monitoring and early intervention. Simultaneously, interventions targeting telomere maintenance or cellular senescence pathways might emerge as novel therapeutic strategies for RA-ILD in the future.

### 5.4. Multi-Biomarker Panels

Given the complexity of RA-ILD pathogenesis, single biomarkers are likely insufficient. Researchers are therefore exploring multi-biomarker panels for diagnosis and risk stratification [35]. A cross-sectional study involving 2001 RA patients identified distinct peripheral blood biomarker profiles associated with RA-ILD by measuring various markers in serum or plasma, including autoantibodies, pro-inflammatory cytokines/chemokines, adipokines, alarmins, and matrix metalloproteinases [88]. These biomarker profiles encompassed multiple biological themes, including innate immunity and allergic response, autoantibodies (such as ACPA, RF, and anti-MAA antibody), adipokines, alarmins, tissue remodeling, and neutrophil chemotaxis. The results demonstrated that models incorporating these principal components (PCs), as well as models combining PCs and MUC5B genotype, were both significantly superior to a model relying solely on clinical risk factors in identifying RA-ILD. This indicates that multi-biomarker panel analysis can enhance the ability to identify RA-ILD and suggests that the pathogenesis of RA-ILD involves diverse pathological pathways.

### 5.5. Summary and Future Perspectives

**Figure 1:**
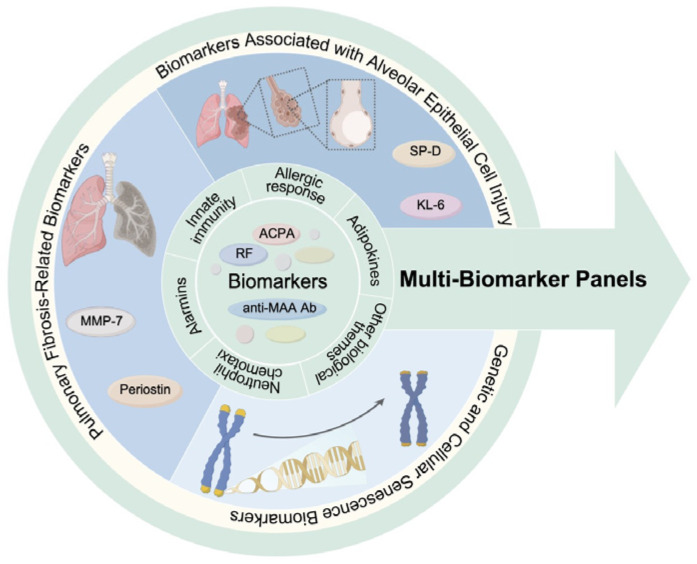
Schematic illustration of biomarker profiles for RA-ILD diagnosis. This includes traditional serum and emerging biomarkers. As single biomarkers are often inadequate, future applications may favor multi-biomarker panels that form unique RA-ILD signatures, potentially involving innate immunity, allergic response, autoantibodies, adipokines, alarmins, tissue remodeling, and neutrophil chemotaxis. Figure 1 was created by Figdraw (https://www.figdraw.com; accessed on 17 November 2025).

The discussed biomarkers show diagnostic value, but their specificity and predictive value require further validation [106]. The diagnostic power of a single biomarker is likely limited. Future efforts should focus on integrating multi-omics data (proteomics, genomics, metabolomics) with clinical and imaging features using advanced computational methods (e.g., machine learning) to develop comprehensive predictive models for accurate diagnosis and risk stratification [91]. Figure 1 schematically illustrates this comprehensive biomarker profile for RA-ILD diagnosis, encompassing both traditional serum biomarkers and emerging biomarkers.

## 6. Conclusions and Future Perspectives

The diagnosis of RA-ILD is evolving from fragmented assessment toward a cohesive, multi-parametric framework. This review synthesizes compelling evidence that early detection and accurate prognostication are unachievable through any single modality. Instead, they depend critically on the concurrent integration of three pillars: robust clinical risk stratification to guide targeted screening, quantitative imaging phenotyping to objectively define disease burden and pattern, and multiplexed biomarker profiling to illuminate the underlying biological activity. This synergistic paradigm directly addresses the core challenge of disease heterogeneity, aiming to reorient clinical management from a reactive to a proactive and personalized mode.

Future research should focus on several key areas: First, integrating multi-dimensional data (genomics, transcriptomics, proteomics, metabolomics, radiomics) using systems biology and network pharmacology to gain deeper pathogenetic insights and discover specific biomarkers and therapeutic targets. Building on this data-driven approach, a key area will involve leveraging AI technologies to process and analyze vast amounts of clinical, imaging, and omics data to develop intelligent diagnostic and risk prediction tools. Furthermore, researchers aim to implement precise subtyping and risk stratification of RA-ILD based on patients’ genetic background, clinical phenotypes, biomarker profiles, and imaging characteristics, to formulate individualized screening and diagnostic plans. Concurrently, close collaboration between rheumatologists, pulmonologists, radiologists, pathologists, and basic scientists is essential to advance diagnostic standards. Finally, strengthening the translation of basic research findings into clinical applications and conducting high-quality randomized controlled trials and real-world studies will be essential to provide evidence-based support for new diagnostic methods. In summary, RA-ILD diagnostic research is advancing rapidly. With continued technological progress and a deeper disease understanding, future RA-ILD diagnosis is expected to become earlier, more precise, and more personalized, ultimately improving clinical outcomes and patient quality of life.

## Figures and Tables

**Table 1:** Main HRCT Manifestation Characteristics and Differential Diagnosis Points of RA-ILD.

HRCT Manifestation	Main Characteristics	Differential Diagnosis	References
UIP Pattern	Reticular opacities, traction bronchiectasis/bronchiolectasis, honeycombing, predominantly subpleural and basal	Idiopathic pulmonary fibrosis, chronic hypersensitivity pneumonitis, NSIP	[39,47]
NSIP Pattern	Ground-glass opacities, fine reticulation, diffuse or basal distribution, relatively symmetrical	Hypersensitivity pneumonitis, UIP, drug-induced lung injury	[39,47,48]
OP Pattern	Consolidation, air bronchogram, subpleural or peribronchial distribution	Cryptogenic organizing pneumonia, infection	[47,48]
LIP Pattern	Ground-glass opacities, centrilobular nodules, thin-walled cysts, diffuse	Lymphoproliferative disorders, Sjögren’s syndrome	[39,47]

Abbreviations: UIP, usual interstitial pneumonia; NSIP, nonspecific interstitial pneumonia; OP, organizing pneumonia; LIP, lymphoid interstitial pneumonia.

**Table 2:** Application of Pulmonary Function Tests in the Diagnosis and Management of RA-ILD.

Aspect of Application	Key PFT Parameters	Typical Findings in RA-ILD	Clinical Significance & Notes	References
Screening & Diagnosis	DLCOFVC	- Reduced DLCO: DLCO serves as an independent marker for detecting ILD in patients with RA, with its decline potentially preceding the manifestation of overt abnormalities on HRCT. - Reduced FVC: Suggests underlying restrictive ventilation dysfunction. Notably, the rate of FVC decline may correlate with specific autoantibody profiles, such as high-titer ACPA.	PFTs are important non-invasive tools for diagnosing RA-ILD, and combining them with other non-invasive methods can improve the early diagnostic rate of RA-ILD.	[21,65,66,67,68]
Severity Assessment & Monitoring Progression	FVC DLCO	Dynamic changes in FVC and DLCO serve as key physiological indicators for predicting prognosis. A decline in FVC of ≥10% or a decrease in DLCO of ≥15% within one year both indicate disease progression and reduced survival rates in patients with RA-ILD.	These specific thresholds of lung function decline are crucial for guiding treatment decisions in RA-ILD, such as initiating or escalating anti-fibrotic therapy.	[65,69]
Correlation with Imaging	FVC TLC DLCO	The extent of certain HRCT findings, such as reticular opacity and honeycombing, is negatively correlated with FVC, TLC, and DLCO, and this association is particularly strong in the UIP pattern of RA-ILD.	PFTs provide functional data that complements the structural information from HRCT, offering a more comprehensive assessment.	[66,70,71]
Characteristic PFT Pattern	FEV1/FVC ratio TLC DLCO	- Restrictive ventilation dysfunction: Reduced FVC with a normal or elevated FEV1/FVC ratio. Reduced TLC. - Decreased diffusion function: Significantly reduced DLCO.	This pattern is the hallmark of RA-ILD and helps differentiate it from obstructive lung diseases.	[47,72,73]

Abbreviations: RA-ILD, rheumatoid arthritis-associated interstitial lung disease; PFTs, pulmonary function tests; HRCT, high-resolution computed tomography; DLCO, diffusion capacity of the lung for carbon monoxide; FVC, forced vital capacity; TLC, total lung capacity; FEV1/FVC ratio, forced expiratory volume in the 1st second/forced vital capacity ratio.

**Table 3:** Comparative Analysis of Emerging Biomarkers for RA-ILD Diagnosis and Assessment.

Biomarker Category	Example(s)	Reported Diagnostic Performance (AUC)	Major Limitations/Current Challenges	Current Evidence Landscape	Key Supporting References
Alveolar Epithelial Injury	KL-6, SP-D	KL-6: 0.939; SP-D: 0.803	- Elevated in other ILDs (e.g., IPF, hypersensitivity pneumonitis) and severe infections, reducing specificity for RA-ILD. - Cut-off values not standardized, influenced by age and ethnicity. - Limited data on guiding treatment decisions.	Primarily from observational studies. Multiple cross-sectional and cohort studies confirm association with RA-ILD and correlation with disease severity/progression.	[66,76,77,78]
Pulmonary Fibrosis	MMP-7, Periostin	MMP-7: Consistently elevated in meta-analysis; periostin: Data emerging, specific AUCs for RA-ILD not yet robustly established.	- Also elevated in IPF and other fibrotic conditions. - Periostin levels can be influenced by atopy and other inflammatory states. - Longitudinal data on their utility for monitoring treatment response is scarce.	Evidence varies in maturity. MMP-7 is supported by a systematic review/meta-analysis; periostin’s association with fibrosis is suggested by preliminary clinical studies, but diagnostic performance data are limited and lack large-scale validation.	[15,76,79,80,81,82,83,84]
Genetic & Cellular Senescence	TL	TL: Shorter TL associated with RA-ILD presence and progression.	- Measurement of TL is complex and not standardized for clinical use. - Peripheral blood TL may not fully reflect lung tissue telomere status.	Evidence for TL’s association with disease comes from multiple cohorts, but methodological standardization and clinical translation require refinement.	[85,86,87]
Multi-Biomarker Panels	Combinations of autoantibodies, cytokines, MMPs, adipokines	Superior to clinical factors alone (AUC: 0.739–0.751 vs. 0.630).	- Technically complex and costly. - Lack of standardized, validated panels. - Requires sophisticated analytical methods (e.g., machine learning). - Clinical translation and practicality are major hurdles.	Proof-of-concept stage. Initial cross-sectional studies demonstrate potential superiority over clinical models, but they lack independent cohort validation and standardized panels have not been established.	[88]

Abbreviations: RA-ILD, rheumatoid arthritis-associated interstitial lung disease; HRCT, High-Resolution Computed Tomography; ILD, Interstitial Lung Disease; IPF, Idiopathic Pulmonary Fibrosis; KL-6, Krebs von den Lungen-6; MMP-7, Matrix Metalloproteinase-7; SP-D, Surfactant Protein D; TL, Telomere Length.

## Data Availability

No new data were created or analyzed in this study.

## Abbreviations

The following abbreviations are used in this manuscript:

ACPA	Anti-Citrullinated Protein Antibody
AI	Artificial Intelligence
AUC	Area Under the receiver operating characteristic Curve
BAL	Bronchoalveolar Lavage
CI	Confidence Interval
CRP	C-Reactive Protein
CTD-ILD	Connective Tissue Disease-associated Interstitial Lung Disease
DLCO	Diffusion Capacity of the Lung for Carbon Monoxide
DLR	Deep Learning Reconstruction
ERS	European Respiratory Society
EULAR	European Alliance of Associations for Rheumatology
FVC	Forced Vital Capacity
HRCT	High-Resolution Computed Tomography
ILD	Interstitial Lung Disease
IPF	Idiopathic Pulmonary Fibrosis
KL-6	Krebs von den Lungen-6
LDH	Lactate Dehydrogenase
LIP	Lymphoid Interstitial Pneumonia
LUS	Lung Ultrasound
MDHAQ	Multidimensional Health Assessment Questionnaire
MDT	Multidisciplinary Team
MMP-7	Matrix Metalloproteinase-7
NSIP	Nonspecific Interstitial Pneumonia
OP	Organizing Pneumonia
OR	Odds Ratio
PFTs	Pulmonary Function Tests
RA	Rheumatoid Arthritis
RA-ILD	Rheumatoid Arthritis-associated Interstitial Lung Disease
RF	Rheumatoid Factor
SASP	Senescence-Associated Secretory Phenotype
SP-D	Surfactant Protein D
TERF1	Telomeric Repeat-Binding Factor 1
TL	Telomere Length
TGF-β1	Transforming Growth Factor-Beta 1
UIP	Usual Interstitial Pneumonia
α-SMA	Alpha-Smooth Muscle Actin
